# Mesenchymal Stromal Cells: Emerging Roles in Bone Metastasis

**DOI:** 10.3390/ijms19041121

**Published:** 2018-04-09

**Authors:** Nicola Graham, Bin-Zhi Qian

**Affiliations:** 1 Centre for Reproductive Health, Queens Medical Research Institute, University of Edinburgh, Edinburgh EH16 4TJ, UK; nicola.graham@ed.ac.uk; 2 Edinburgh Cancer Research UK Centre, University of Edinburgh, Edinburgh EH4 2XR, UK

**Keywords:** bone, metastasis, tumor microenvironment, stromal cells, mesenchymal stem cells, cancer-associated fibroblasts, metastatic niche, dormancy

## Abstract

Bone metastasis is the most advanced stage of many cancers and indicates a poor prognosis for patients due to resistance to anti-tumor therapies. The establishment of metastasis within the bone is a multistep process. To ensure survival within the bone marrow, tumor cells must initially colonize a niche in which they can enter dormancy. Subsequently, reactivation permits the proliferation and growth of the tumor cells, giving rise to a macro-metastasis displayed clinically as a bone metastatic lesion. Here, we review the evidences that suggest mesenchymal stromal cells play an important role in each of these steps throughout the development of bone metastasis. Similarities between the molecular mechanisms implicated in these processes and those involved in the homeostasis of the bone indicate that the metastatic cells may exploit the homeostatic processes to their own advantage. Identifying the molecular interactions between the mesenchymal stromal cells and tumor cells that promote tumor development may offer insight into potential therapeutic targets that could be utilized to treat bone metastasis.

## 1. Introduction

Metastasis is a major challenge in oncology clinics that contributes to 80% of cancer-associated deaths. Bone is the most common metastatic site for many cancers, including breast, prostate, and lung cancers, with approximately 70% of patients with advanced disease exhibiting bone metastasis [1,2,3]. Patients with bone metastasis not only experience substantial morbidity such as pain, increased risk of fracture, and hypercalcemia, but also exhibit reduced a 5-year survival rate of 26% and 33% in breast and prostate cancer, respectively [4]. While palliative treatments such as anti-osteolytic bisphosphonates are available to improve such symptoms and lessen the morbidity associated with bone metastasis, these do not significantly enhance survival. Bone metastases are often resistant to anti-tumor treatments and therefore there remains no cure [5].

Tumors have previously been described as a “wound that does not heal” displaying many features similar to the wound healing response. These include the infiltration of immune cells and mesenchymal stromal cells, vasculature, and non-cellular components such as the extracellular matrix, which together make up the tumor microenvironment (TME). It is now evident that the TME plays an important role in tumor development by establishing interactions between these host components and the tumor cells [6]. One important component of the TME is mesenchymal stromal cells, which comprise mesenchymal stem cells (MSCs), pericytes, fibroblasts, and osteoblasts. These stromal cells have been shown to promote tumor development, metastasis, and therapy resistance through several pro-tumorigenic effects including: enhanced tumor growth via growth factor release and stimulation of angiogenesis; promoted migration and invasion by the induction of the epithelial-to-mesenchymal transition and production of matrix metalloproteinases (MMPs); and immune evasion via interactions with the immune cells to create an immunosuppressive environment [7,8,9]. However, this research is mostly limited to the primary tumor.

Bone metastatic cancers often have already spread at the time of diagnosis, with disseminated tumor cells (DTCs) being detected in the bone of many patients. These DTCs are drug resistant and can give rise to secondary bone metastasis years after the initial resection or treatment of the primary tumor [10]. This suggests that the pro-tumorigenic effects of the mesenchymal stromal cells within the primary tumor may have already occurred before initial diagnosis; therefore, it may be more appropriate to therapeutically target the DTCs at the secondary site rather than prevent the dissemination from the primary tumor in the first place. This review will therefore focus on the role of the mesenchymal stromal cells within secondary bone metastasis after the tumor cells have reached the site. Initially the mesenchymal stromal cells contribute to a niche that facilitates homing and colonization. Within this niche, the tumor cells can survive and remain dormant, and may eventually reactivate and grow to establish a metastatic lesion within the bone. We will discuss the molecular mechanisms that regulate these processes and highlight potential therapeutic targets that may serve as a way to combat bone metastasis in the clinic.

## 2. Mesenchymal Stromal Cells within the Tumor Microenvironment

**Figure 1:**
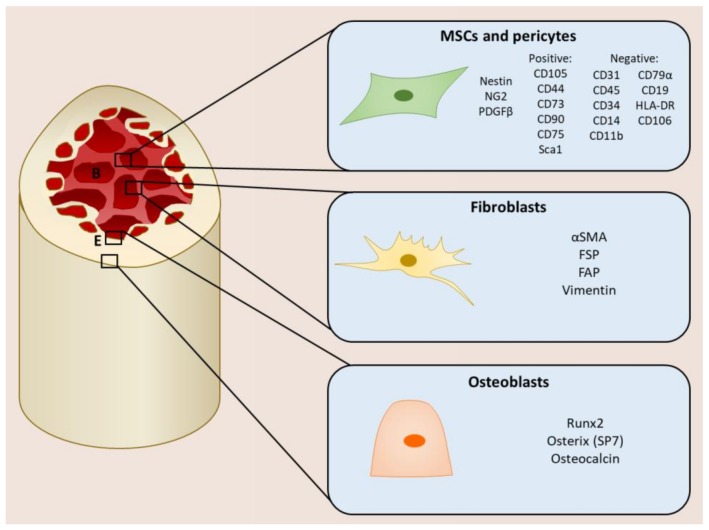
Mesenchymal stromal cells within the bone. MSCs, pericytes and fibroblasts are found at the perivascular niche along the blood vessels (B), whereas osteoblasts are located at the endosteal niche (E). (NG2 neural glial antigen 2; PDGFβ platelet derived growth factor beta; αSMA alpha smooth muscle actin; FSP fibroblast specific protein; FAP fibroblast activation protein) [5,9,13,14,15,26].

The mesenchymal stromal compartment of the TME consists of MSCs, pericytes, fibroblasts, and osteoblasts, which are also found in different regions of the bone and can be defined by different cell markers (Figure 1). MSCs are multipotent cells that play a role in tissue maintenance and the regeneration of connective tissues including bone, cartilage, and adipose tissue by differentiating into osteoblasts, chrondocytes, and adipocytes, respectively [7,8]. They are also recruited to wounds during repair, where they produce extracellular matrix (ECM) proteins and secrete cytokines that promote the recruitment of immune cells [11]. Within the bone, MSCs are a rare population, making up about 0.001–0.01% of total cells. Here, they not only contribute to bone turnover by differentiating into bone-producing osteoblasts, but also provide a perivascular and endosteal compartment that maintains the hematopoietic stem cells (HSCs), known as the HSC niche [12,13]. Since they were first identified by Friedenstein over 40 years ago, the true lineage and identification of MSCs remains controversial due to the lack of a specific marker. Currently MSCs are defined by several characteristics in vitro:

- Adherence to plastic;
- Ability to self-renew;
- Ability to differentiate into osteoblasts, chrondocytes, and adipocytes;
- Surface marker expression (Figure 1) [14].

Bone marrow MSCs have been defined by their expression of Nestin [15,16], CXCL12 [17,18], and neuron-glial antigen 2 (NG2) [13,16] in different studies, and share many characteristics with other MSC-like cells, such as pericytes and CXCL12-abundant reticular (CAR) cells. Pericytes are also located at the perivascular region and are important for maintaining vessel integrity. They are classically defined by the expression of NG2 and platelet-derived growth factor receptor beta (PDGFRβ), and exhibit similar features to those that define MSCs [13]. This makes it difficult to ascertain the exact cell identity, which is further complicated by their multiple tissues of origin. It may be possible that they are subpopulations of MSC-like cells or different stages of development or differentiation.

Fibroblasts are also a significant component of the TME and are associated with metastasis and poor prognosis [19]. In normal conditions, fibroblasts can be considered as inactive cells; however, during inflammatory responses, such as wound healing and fibrosis, fibroblasts are activated by becoming myofibroblasts and acquiring the expression of alpha smooth muscle actin (αSMA), fibroblast activated protein (FAP), and fibroblast specific protein (FSP). Myofibroblasts mediate the remodeling of the ECM by secreting MMPs to degrade the existing ECM as well as depositing new components such as collagens, laminins, and fibronectin. They also recruit immune cells to the wound and promote angiogenesis through the secretion of cytokines [9,20,21]. Cancer-associated fibroblasts (CAFs) exhibit an activated phenotype similar to myofibroblasts [22]. However, whilst the deactivation and removal of myofibroblasts is coordinated with the resolution of the wound, CAFs remain in a permanently activated state and continue to secrete factors involved in tumorigenesis, including ECM factors and cytokines [23,24,25].

Another type of mesenchymal stromal cell abundant in the bone marrow that plays a role in bone metastasis is osteoblasts [27]. Osteoblasts originate from the terminal differentiation of MSCs regulated by transcription factors Runt-related transcription factor 2 (Runx2) and osterix, and are a key player in bone turnover and remodeling. Osteoblasts contribute to bone production by secreting osteoid, which is mineralized to form new bone. They also regulate the activity of osteoclasts, which are responsible for bone resorption: the secretion of osteoblast-derived receptor activator of nuclear factor kappa-B; ligand (RANKL) activates osteoclasts, whereas the secretion osteoprotegerin (OPG), which inhibits RANK/RANKL interaction, prevents osteoclastogenesis and subsequently bone resorption [5,26].

Cancer-associated mesenchymal stromal cells evolve from several mechanisms including recruitment from other tissues, the activation of resident cells, and the differentiation of MSCs to fibroblasts and osteoblasts, as well as other sources such as the epithelial-to-mesenchymal transition or differentiation from other cells types, for example smooth muscle and endothelial cells [28]. MSCs are recruited to and persist in tumors in several models of primary cancers [29,30,31,32] due to the secretion of tumor-derived chemoattractants such as CXCL12, CXCL16, LL37, and cyclophilin B [33,34,35,36]. Similar molecular interactions may serve as potential chemoattractants to induce MSC recruitment to bone metastasis. Both bone and bone metastasis secrete many cytokines such as vascular endothelial growth factor (VEGF) [37,38,39] and CXCL12 [40], for which MSCs express receptors [41]. The bone marrow and bone metastasis are also hypoxic [42,43,44,45], and may induce MSC recruitment to bone metastatic lesions, as demonstrated in ischemia models [46]. MSCs migrate towards breast cancer cells via hypoxia-induced interleukin 6 (IL6) secretion [47] and towards primary breast tumors via the hypoxia-inducible factor alpha (HIFα)-dependent secretion of CXCL16 [48]. Hypoxia-induced hepatocyte growth factor (HGF) may also mediate recruitment [49] as it is upregulated in serum of breast cancer patients [50,51] and associated with tumor formation and metastasis in breast [52,53] and prostate cancer [54]. Furthermore, the activation of HGF receptor, MET, in bone marrow stromal cells by melanoma cells is associated with metastasis [55].

**Figure 2:**
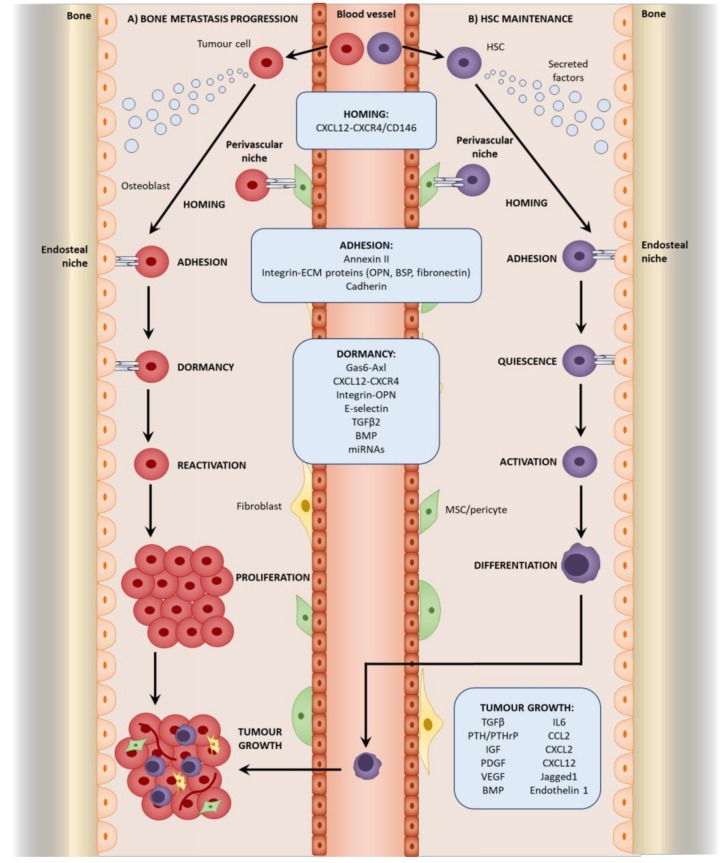
The involvement of the hematopoietic stem cell (HSC) niche in (**a**) bone metastasis progression and (**b**) HSC maintenance. Disseminated tumor cells (DTCs) and HSCs home to the endosteal and perivascular where they adhere to the osteoblasts and mesenchymal stem cells (MSCs), respectively, and remain dormant or quiescent. The tumor cells can then be activated and begin proliferating to form a bone metastatic lesion, whereas the HSCs differentiate to give rise to immune cells that enter the circulation (OPN osteopontin; BSP bone sialoprotein; TGFβ transforming growth factor β; PTH parathyroid hormone; PTHrP parathyroid hormone-related protein; IGF insulin growth factor; PDGF platelet derived growth factor; VEGF vascular endothelial growth factor; BMP bone morphogenetic protein; IL6 interleukin 6; CCL2 C-C motif chemokine 2; CXCL12 C-X-C motif chemokine 12) [5,27,72,76,78,80,82,92,93,94,95,96,97,98,99,100,101,102,103,104,105,106,107,108,109,110,111,112,113,114,115,116,117,118,119].

Cancer-associated mesenchymal stromal cells also exhibit an activated phenotype. The exposure of MSCs, osteoblasts, and fibroblasts to tumor cells and tumor-derived factors induces an “activated” phenotype that exerts more pro-tumorigenic effects than “naïve” cells [20,24,25,56,57,58,59]. CAFs can also arise from the tumor-induced differentiation of MSCs and pericytes [33,60,61]. Tumor-associated MSCs also differentiate into osteoblasts as demonstrated in breast cancer lung metastasis. Interestingly, MSCs recruited to subcutaneous breast tumors differentiated into adipocytes, indicating the role of the environmental cues in differentiation [31]. However, most studies investigate the origins of mesenchymal stromal cells in primary tumors, with research into the origin at secondary sites being limited to breast cancer lung and liver metastasis. Research into the origins of mesenchymal stromal cells in bone metastasis awaits much-needed research. Nevertheless, it is evident that they play a role throughout the establishment of bone metastasis (Figure 2).

## 3. Mesenchymal Stromal Cells in the Colonization of the Bone

One of the first pro-tumorigenic effects of mesenchymal stromal cells within bone metastasis is the facilitation of seeding and colonization. The majority of DTCs reaching the bone do not survive, nor give rise to metastatic lesions [62,63]. In order to persist, DTCs may colonize specialized niches that promote their survival. The colonization of the bone by tumor cells is a multistep process where the circulating tumor cells (CTCs) initially home to the bone, and seed within the metastatic niche. It is believed that a pre-metastatic niche is initially formed before the arrival of the tumor cells, mediated by signaling from primary tumor, and provides a favorable microenvironment for the tumor cells. Mesenchymal stromal cells may be a significant component of these pro-tumorigenic niches and contribute to the colonization process by establishing molecular interactions with the tumor cells. These not only retain the tumors cells at the niche but are also thought to promote the survival and dormancy of the tumor cells.

### 3.1. Metastatic Niches within the Bone

Steven Paget’s “seed and soil” theory was a key landmark in investigating metastasis. It hypothesized that tumor cells, or the “seed”, can only metastasize to a receptive microenvironment, or “soil” [64]. The formation of a pre-metastatic niche is therefore important in metastasis, and whilst the study of the pre-metastatic niche in the bone is lacking, research on lung metastasis may offer insight into potential mechanisms. Resident lung fibroblasts are activated by thrombospondin-2 released from CTCs and form a pre-metastatic niche to mediate colonization in lung metastasis of breast cancer [65]. Within the lungs of mice implanted with Lewis lung carcinoma and B16 melanoma cells, resident fibroblasts also deposit fibronectin in response to factors secreted from the tumor cells. This promotes the recruitment and adhesion of bone marrow-derived hematopoietic progenitor cells (HPCs) via integrin α4β1-fibronectin interactions before the arrival of tumor cells, creating a permissive pre-metastatic niche that facilitates colonization. VEGFR1 inhibition or depletion of VEGFR1^+^ HPCs was found to prevent the formation of the pre-metastatic niche and subsequent metastasis [66]. A similar interaction between fibronectin and integrin α4β1 exists within the bone and is important in the homing of chronic lymphocytic leukemia B cells to stromal cells in the bone marrow [67]; therefore, similar effects of fibroblast-derived fibronectin and HPC recruitment in pre-metastatic niche formation may occur during bone metastasis. Within the bone, the HSC niche responsible for maintaining HSCs [68] may be utilized by malignant cells as a niche for their metastasis. Two HSC niches have mainly been identified within the bone: the endosteal niche comprising predominantly of osteoblasts promotes HSC quiescence [69,70] and is mediated by interactions involving parathyroid hormone-related protein (PTHrP) and bone morphogenetic protein (BMP) [71,72,73]; the perivascular niche comprising of MSCs/pericytes regulates HSC proliferation and mobilization [15,16,17,18], (reviewed by [74]). The existence of these pre-formed HSC niches may be a reason for the favorable tropism of cancers such as breast, prostate, lung, and thyroid to the bone [3] as there is no dependence on the initial establishment of the pre-metastatic niche [75]. As CTCs invade the bone, they home to the HSC niche where they adhere to the osteoblasts and MSCs. Here, they may compete with the HSCs and utilize the similar molecular interactions that exist between the HSCs and stromal cells located within the niche. For this reason, tumor cells have been referred to as a “molecular parasite” that can exploit resources from the niche (Figure 2) [76].

The first direct evidence that tumor cells utilize the HSC niche during the colonization of the bone was demonstrated by Shiozawa et al. [76] using a model of prostate cancer. Disseminated prostate cancer cells localized to the endosteal niche and competed with the HSCs for occupancy of the niche. The presence of DTCs within the bone reduced HSC engraftment to the niche after bone marrow transplantation, and mobilization of the HSCs from the niche resulted in increased DTCs, suggesting that DTCs were occupying the niche. Interestingly, these observations were specific to metastatic prostate cancer cells as non-metastatic cell lines did not prevent HSC engraftment after transplantation [76]. This study is supported by the specific homing and occupancy of the osteoblast-rich endosteal niche during bone colonization in breast cancer models [77,78,79,80]. Colonization may be dependent on a niche formed from specifically senescent osteoblasts. A conditional mouse model that induced senescence in the osteoblasts was developed by stimulating the expression of cell-cycle inhibitor p27^kip1^ in osteoblasts using a stromal-specific, estrogen-responsive Cre recombinase. After intracardiac injection into the circulation of these mice, breast cancer cells localized to senescent osteoblasts and resulted in increased tumor burden compared to littermate controls. This tumor burden was also correlated with the number of senescent osteoblasts, suggesting that they form a niche to support the seeding and growth of bone metastasis [81]. Recently, it was demonstrated that melanoma cells also home to the perivascular niche. The depletion of MSCs/pericytes in PDGF-β knockout mice resulted in reduced bone metastasis burden [82]. Moreover, parathyroid hormone (PTH), which is increased in bone metastasis, also enhances MSC proliferation and causes HSC expansion, and therefore may enlarge the perivascular niche [15]. Within the perivascular niche, the nestin^+^ MSCs also crosstalk with the CD169^+^ macrophages, therefore the seeding may not be dependent only on the MSCs [83].

It is evident that primary tumors or CTCs can modulate the HSC niche in preparation for metastasis by the secretion of factors into the circulation that act on the mesenchymal stromal cells. The activation of osteoblasts by primary tumor-derived factors enhanced bone remodeling before the arrival of the tumor cells, which promoted the colonization of the tumor cells and the establishment of bone metastasis [84,85]. This is further supported by the association of breast cancer micrometastasis with increased osteoblasts before the establishment of clinical metastasis [86]. Several tumor-derived molecules have been demonstrated to enhance osteoblast activity, including primary prostate cancer-derived BMP6 [84]. Disseminated prostate cancer cells also indirectly induce MSC to osteoblast differentiation and enhance osteoblast activity by secreting IL6, which stimulates BMP2 and BMP6 from HPCs [85]. BMPs also regulate VEGF secretion from prostate cancer cells [87], which may also promote colonization via enhanced osteoblast activity [87,88]. Conditional media from prostate cancer cells induced osteoblast differentiation, which was attenuated by a VEGF receptor inhibitor. However, other factors play a role as VEGFR inhibition only partially blocked differentiation and treatment with VEGF was not sufficient to induce osteoblast differentiation. Nevertheless, the treatment of bone metastasis-bearing mice with VEGFR inhibitor resulted in reduced bone remodeling and tumor burden. Notably, this was associated with reduced bone remodeling only in the presence of bone metastasis, and not in normal physiological bone turnover, suggestive of a tumor-specific mechanism that thus may be more targetable [88]. Bone metastatic breast cancer cells also exhibit increased VEGF [89], which may have similar effects on colonization. Specific isoforms of VEGF may also be responsible for enhancing colonization. Breast cancer cells overexpressing VEGF189 delayed and decreased colonization in both bone and lungs and was associated with increased αSMA^+^ stromal cells compared to cells overexpressing VEGF165, which exhibited metastasis rates similar to control cells [90]. Identifying the endogenous isoform involved in enhancing bone metastasis may also allow for more specific therapeutic targeting. Osteoblasts are also activated by a soluble receptor for advanced glycation end products (sRAGE) released from primary lung adenocarcinoma, and whilst this results in the recruitment of tumor-promoting neutrophils to the primary tumor, it would be interesting to investigate the effect of these activated osteoblasts during the bone colonization of lung cancer [59]. PTH, which is increased in cancer patients [91], also increases the number of osteoblasts [71] and therefore expands the osteogenic niche. The treatment of mice with PTH prior to intracardiac injection of breast cancer cells to establish bone metastasis induced an increase in osteoblasts, but was not associated with increased colonizing DTCs [27]. Furthermore, the secretion of PTHrP from prostate cancer modulated osteoblasts, including CCL2 upregulation. This promoted osteoclast-mediated bone resorption, which also created a favorable metastatic niche for the prostate cancer cells [92].

### 3.2. Homing to the Niche

Prior to seeding, CTCs must first home to the bone, which is mediated significantly using the CXCL12-CXCR4 pathway [76], one of the key interactions between HSCs and the niche [98]. CXCL12 from both the osteoblasts and MSCs at the endosteal and perivascular niches, respectively, interacts with CXCR4 and CD146 to induce the migration of CTCs towards the niches, and mediates the extravasation and homing of the CTCs to the bone [76,82]. The silencing of CXCL12 and CD146 in their respective cells resulted in reduced extravasation and thus attenuated metastasis [82]. The role of CXCL12 in homing to the bone is further supported by in vitro migration assays that demonstrated that the breast and prostate cancer cells migrate towards CXCL12 and osteoblasts, which is dependent on CXCR4 [120,121,122]. Interestingly, both radiation and chemotherapeutic agents (cyclophosphamide and 5-florouracil) increase CXCL12 expression from the endosteal niche, thus similar anti-cancer treatments in the clinic may enhance the homing of the tumor cells to the bone, and therefore promote bone metastasis [123]. Moreover, the activation of CXCR4 can regulate VEGF expression [124,125,126]. Therefore, CXCL12-CXCR4 interactions during homing to the HSC niche may stimulate VEGF secretion from prostate cancer cells. This may result in a further expansion of the osteogenic niche, establishing a positive feedback loop by allowing an increased capacity for tumor cell homing to the niche.

In a model of prostate cancer bone metastasis, CXCL12 expression was increased in the bone with the strongest staining observed within the endosteal region associated with osteoblasts [127], similar to osteoblast-derived CXCL12 in HSC homing [71]. Furthermore, CXCL12 is not secreted in stromal cells of other tissues that are not common sites of metastasis for cancers associated with bone metastasis [120]. A similar expression pattern was also observed in breast cancer, where CXCL12 was expressed in tissues commonly affected by breast cancer metastasis including the bone marrow, liver, lung, and lymph nodes, but not other tissues such as the kidney and small intestine [128]. Moreover, CXCL12 expression was found to be higher in breast cancer bone metastasis compared to other sites of metastasis [40]. This positive correlation between CXCL12 expression and sites of metastasis support the role the CXCL12-CXCR4 axis in selective homing and the colonization of bone, and may explain the tropism of cancers towards the bone [120,128]. CXCR4 is also elevated in prostate, breast, and melanoma cancer cells and tissue compared normal samples, and is associated with tumor stage, a metastatic phenotype, and poor survival [120,122,128,129,130,131]. Furthermore, bone metastatic breast cancer cells selected via successive injections and recovery from the bone display upregulated CXCR4 compared to the parental cell lines, suggesting that bone metastatic tumor cells acquire the expression of CXCR4 that facilitates the homing towards the CXCL12 at the niche [113]. Bone metastatic tumor cells may also be selected for in the primary tumor, in which stromal-derived CXCL12 selects for clones that are able to home to and survive in the CXCL12-rich region of the bone marrow [40]. The increased CXCR4 expression on bone metastatic cells compared to non-metastatic cells [113,128] may enhance tumor cell association with the niche in a similar manner to HSC homing to the niche, in which higher CXCR4 expression is associated with more rapid engraftment and reconstitution [132]. The overexpression of CXCR4 in breast cancer cells resulted in increased bone metastasis formation [113], whereas CXCR4 inhibition significantly reduced metastatic burden in an experimental model of prostate cancer bone metastasis [127]. The use of AMD3100, a CXCR4 partial agonist, also caused the mobilization of prostate cancer cells from the niche back into the circulation and may be therapeutically relevant [133]. Whilst Shiozawa et al. [76] demonstrated that CXCR4 inhibition decreased bone marrow prostate cancer cells by inducing the mobilization of the tumor cells back into the circulation, Wang et al. [134] showed that the same treatment regime did not affect the number of prostate DTCs that homed to the bone, but instead altered the distribution. The prostate cancer cells preferentially colonized to lateral endocortical regions of the bone where osteoblasts and bone formation are increased in comparison to the medial side, and was disrupted during CXCR4 inhibition. The difference between these two studies may be due to the fact that latter identified the time point at which the tumor cells left or re-entered the bone marrow. It may also be due to the involvement of additional molecules in homing to the bone.

The role of other factors in homing to the bone is further supported by the partial inhibition of prostate cancer bone metastasis after the blockade of CXCR4. CXCR7 is an alternative receptor for CXCL12 [135,136], and is also increased in prostate cancer tissues displaying correlations with tumor stage and bone metastases. CXCR4 and CXCR7, both receptors for CXCL12, may reciprocally regulate each other and provide alternative mechanisms or redundancy for homing to the niche, which is of importance to therapeutic targeting [135,136]. The utilization of CXCL12-CXCR4 during homing may also be difficult to target due the overlap in roles between tumor cell homing and HSC homing, as targeting DTCs using CXCR4 inhibition would also release HSCs from the niche, causing significant clinical adverse effects [133].

Other targetable interactions between the mesenchymal stromal cells and cancer cells also mediate homing to the bone. The use of ex vivo explants to image homing and colonization revealed that breast cancer cells seeded directly with bone tissue fragments infiltrated the marrow compartment and homed to the stromal cells, which was associated with increased IL1β and leptin [78,137,148]. CXCL16-CXCR6 interactions, which have previously been implicated in prostate cancer [33], may also mediate homing to the bone. CXCL16 induces the migration and invasion of the prostate cancer in vitro via CXCR6 [149], which is increased in prostate [150,151] and breast cancer cells and tumors, and is associated with aggressiveness and tumor stage [149,152]. Furthermore, CXCL16 was expressed in bone metastasis [149,150], with weak staining detected in the lungs and liver metastasis, and was also correlated with tumor stage [152]. This data suggests a role of these chemokine interactions in tumor cell homing and colonization of the bone, but research to confirm their potential role in vivo is required. Other stromal cell-derived chemoattractants that promote tumor cell migration have been identified in primary tumors (Table 1). Many of the studies use bone-derived MSCs, thus the molecular interactions may be relevant and offer insight into factors involved in homing to the bone.

### 3.3. Direct Interactions with the HSC Niche

Once homed to the niche, direct cell-cell interactions between the tumor cells and mesenchymal stromal cells are established to maintain contact at the niche. These interactions are similar to those used by the HSCs [153]. Annexin-II is a phospholipid-binding membrane protein expressed by both osteoblasts and endothelial cells, and mediates HSC adhesion within the niche [154]. Similarly, prostate cancer cells express the annexin-II receptor and utilize annexin-II to migrate and adhere to osteoblasts at the endosteal niche during colonization. Inhibition of both annexin-II and annexin-II receptors reduced adhesion to osteoblasts and the homing of prostate cancer cells to the endosteal niche in vivo and is associated with reduced metastasis [155]. Annexin-II is also expressed by metastatic breast cancer cells [156] and is associated with tumor progression, metastasis [157], and drug resistance [158,159], further supporting its role in metastatic colonization [160]. Furthermore, the pattern of annexin-II expression at the endosteal niche is similar to CXCL12. In annexin-II-deficient mice, as well as a reduced number of HSCs, the expression of CXCR4 and CXCR7 was also reduced. This suggests that annexin II may regulate CXCR4/CXCR7, and therefore crosstalk between CXCL12-CXCR4 and annexin-II may occur during homing and adhesion to the niche [161].

Another molecular interaction involved in HSC maintenance at the endosteal niche and exploited by tumor cells during colonization is cadherins, which maintain adherence between the osteoblasts and HSCs [72,101]. Several types of cadherin have been identified at the HSC niche, including VE-cadherin, OB-cadherin, and N-cadherin [101,162,163,164]. Cadherins are important for tumor cell adhesion and the colonization of bone metastasis at the endosteal niche, and are most strongly evidenced using an experimental model of breast cancer. After intra-iliac injection to establish bone metastasis, tumor adherence to the endosteal niche was mediated by heterotypic junctions formed between N-cadherin on the osteoblasts and E-cadherin on the tumor cells [80], which is associated with poor prognosis [165]. Similar results were also observed in an explant model of breast cancer bone metastasis. A knockout of N-cadherin in the osteogenic lineage or antibody neutralization of E-cadherin in the cancer cells resulted in reduced colonization and subsequent tumor growth [78]. Breast cancer cells also directly bind to osteoblasts via OB-cadherin [166]. OB-cadherin is also elevated in bone metastatic subclones of breast cancer cells in comparison to the parental cells [167] and upregulation in metastatic prostate cancer cells facilitates the adhesion to osteoblasts in vitro. The downregulation of OB-cadherin in metastatic prostate cancer cells reduced their binding and migratory ability [168] and attenuated bone metastasis burden in vivo [169]. In contrast, the overexpression of OB-cadherin in breast cancer cells enhanced bone metastasis due to an increase in early colonization, which appears to be a bone-specific effect as there was no effect on lung metastasis [167]. Together, these results suggest that OB-cadherin interactions between cancer cells and osteoblasts mediate the colonization of the bone at the endosteal niche. OB-cadherin is also expressed by MSCs and fibroblasts, and may also mediate colonization at the perivascular niche [170]. Interestingly, the overexpression of CXCR7, which is often expressed in bone metastatic cells, results in an upregulation of OB-cadherin, therefore interactions between CXCL12-CXCR7 during homing may promote adhesion to the niche by inducing cadherin expression [136].

Integrins are transmembrane proteins that are also important at the endosteal niche. HSCs express integrin α4β1 and β2, which are essential for HSC engraftment and retention within the niche [93,94,95,96] by binding to their respective ligands, vascular cell adhesion protein 1 (VCAM1) and intercellular adhesion molecule 1 (ICAM1) [123]. The expression of α4β1 and β2 in HSCs is activated by stromal- and endothelial-derived CXCL12. Integrin-expressing tumor cells may exploit similar molecular interactions to bind to stromal-derived ECM proteins and maintain retention at the niche. CXCL12 treatment of prostate cancer cells results in increased αvβ3 expression and receptor affinity, which enhances cell adhesion to human bone marrow cells [97] and their ability to bind to the ECM protein vitronectin [99]. This CXCL12-mediated expression of αvβ3 is more prominent in highly metastatic prostate cell lines [97]. It is therefore possible that the binding of CXCR4 receptors to CXCL12 during homing to the HSC niche induces integrin expression on the tumor cells, which mediates subsequent adhesion to ECM proteins secreted by the stromal cells at the niche. Factors in the primary tumor may also select for integrin-expressing tumor cells. In primary breast cancer, Runx2 regulates the expression of integrin α5 on tumor cells and is correlated with the risk of bone-specific metastasis. Integrin α5 mediates the migration and adhesion of breast cancer cells towards osteoblast-like cells and bone deposited from these cells. This suggests that Runx2-mediated integrin α5 expression within the primary tumor selects cells that are more likely to colonize the bone and have a survival advantage at the secondary site [171].

One of the ECM proteins that the integrins may interact with is osteopontin (OPN), which is expressed at the endosteal niche and plays a role in HSC migration and adhesion to the niche [69,100]. Although OPN has no effect on primary tumors [172], OPN is elevated in patients with breast, prostate, and lung cancer [173,174] and is associated with metastasis, recurrence, and poor survival [32,175,176,177,178]. OPN interacts with a variety of integrins including αvβ3, α9v1, αvβ5, αvβ1, α5β1, and α4β7 [179,180,181,182], as well as CD44. Many of these integrins are also increased in prostate and breast bone metastatic cells [99,183] and multiple myeloma cells [184], and are also associated with tumor progression and metastasis [185]. The migratory and adhesive effects of OPN on cancer cells via binding to integrins and CD44 on the tumor cells may mediate colonization to the endosteal niche. The interaction of OPN with CD44 induces the migration of breast, melanoma, and multiple myeloma cells [186,187,188]. Highly metastatic breast cancer cells also migrate towards OPN [178] via the binding of β3 [189], αvβ5, and αvβ1 integrins [190], whereas non-metastatic cells do not express these integrins [189,191] nor migrate towards OPN. Furthermore, the overexpression of αvβ3 integrin in breast cancer resulted in increased metastatic incidence and growth in an experimental model of bone metastasis, whereas αvβ3 inhibition reduced bone metastasis. The injection of these breast cancer cells directly into the tibia had no effect on tumor burden compared to control breast cancer cells, suggesting that its effects on bone metastasis occur early on, such as during colonization [192]. The activation of PTHR via PTHrP treatment, which is often increased bone metastasis [114], enhances the production of OPN from the osteoblasts [100], and therefore may augment adhesion to the endosteal niche, thus promoting colonization. Fibroblasts also serve as a source of OPN [32,193,194], which mediates the adhesion of breast cancer cells in vitro and may influence colonization in the bone marrow [153,195]. The reciprocal interaction also exists where integrins within the niche bind to tumor-expressed OPN [196,197]. OPN-deficient breast cancer cells display a reduced ability to initiate tumors with no effect on growth in both subcutaneous tumors and lung metastasis [198,199]. A similar phenomenon may occur in bone metastasis. Integrins expressed by tumor cells can also bind to other ECM proteins including fibronectin and bone sialoprotein (BSP). The latter is associated with breast cancer bone metastasis [200,201], and mediates the migration of prostate cancer cells by binding α5β1 [202]. Collagen I is another ECM protein expressed by MSCs and binds to prostate cancer cells via integrin receptors α1β1 and α2β1, with a higher affinity observed in metastatic cells compared to non-metastatic cells [203]. Alternatively, the expression of a collagen receptor, discoidin domain receptor family member 1 (DDR1), on lung cancer cells facilitates the colonization of the bone, and is associated with poor survival [204]. Fibroblast-derived tenascin-C is also involved in colonization, as demonstrated in the metastasis of breast cancer to the lung, and may play a similar role in bone metastasis [25].

Once established within the bone, DTCs can either enter dormancy by becoming quiescent, or proliferate to give rise to a metastatic lesion. The signals within the HSC niche, as well as factors from the stromal cells, may mediate these processes.

## 4. Mesenchymal Stromal Cells in Tumor Cell Dormancy

The late recurrence of bone metastasis due to dormant DTCs within the bone marrow is an unmet clinical need. By investigating the molecular interactions between the mesenchymal stromal cells and DTCs that maintain dormancy, new potential therapeutic targets may be identified. Bone metastasis can arise from residual dormant cells years after the primary tumor has been surgically resected or treated, a process defined as relapse or recurrence. At the time of diagnosis, DTCs are detected within the bone marrow of 15.5–30% and 13–72% of breast [10,205,206] and prostate [62,207] cancer patients, respectively. The presence of DTCs within the bone marrow is associated with poor prognosis and reduced disease-free survival [62,205]. This suggests that these DTCs may indeed develop into metastatic lesions. However, a significant proportion of patients do not develop bone metastasis [62,63], which may be due to two potential reasons: the tumor cells require the localization to a niche in which they receive survival or proliferative signals, without which the tumor cells will die in the bone marrow as discussed previously; or, the DTCs enter a latent stage in which they must be reactivated, and without these activation signals the cells remain dormant. The asynchrony between the development of the primary tumor and bone metastasis suggests that DTCs can remain within the bone for many years after the initial treatment to which they are resistant [205,208,209]. Breast cancer cells within the bone marrow are often negative for proliferative markers, which may cause resistance to anti-cancer treatments such as chemotherapy [210,211]. Together, these data suggest that bone-associated DTCs enter dormancy. Dormant DTCs are characterized by their growth arrest, survival in the microenvironment, and resistance to therapy, and therefore may be an important underlying factor in treatment failure [212].

The presence of DTCs at the time of diagnosis indicates that dissemination occurs during the early progression of tumor development. This was demonstrated by Husemann et al. [213], who showed that DTCs and micrometastases were present in the bone marrow of PyMT and Her2 transgenic mice during early stage tumor development, and in wild-type mice transplanted with premalignant mammary glands. These early DTCs were able to give rise to late stage metastases, suggesting they can survive within the bone marrow and eventually develop into bone metastasis [214]. The bone marrow therefore acts as a reservoir for these dormant cells to survive by creating a niche. It is evident that dormant DTCs are influenced by the bone microenvironment. Breast cancer patients treated with zoledronic acid, which inhibits osteoclastogenesis, exhibited a significant reduction of dormant DTCs in the bone marrow with the elimination of DTCs in 87% of patients. This demonstrates that bone marrow plays a role in the maintenance of DTCs and are affected by alterations to this microenvironment [213]. Therefore, signals from the mesenchymal stromal cells within the bone also appear to mediate the dormancy of the DTCs.

### 4.1. Dormancy at the Endosteal Niche

The HSC niche may maintain tumor cell dormancy in an analogous manner to the long-term survival and quiescence of HSCs utilizing similar molecular interactions within the niche (Figure 2) [215,216]. The existence of a dormant niche was first evidenced by Ghajar et al. [102], who demonstrated that non-proliferating dormant disseminated breast cancer cells reside adjacent to the perivascular region in the bone marrow. This was further supported by the localization of dormant cells to the endothelium [78]. To study the role of the perivascular niche in dormancy, breast cancer cells were seeded onto microvascular networks formed in vitro by co-culturing endothelial cells and bone marrow MSCs. This resulted in the reduced proliferation of the cancer cells due to an upregulation of thrombospondin-1 [212], which was previously implicated in suppressing metastatic growth in the lung [217], where it is expressed at the perivascular region in association with dormant DTCs [212]. In vivo studies demonstrated that at the sinusoidal niche, vascular E-selectin mediates the localization of non-proliferating breast cancer cells expressing E-selectin ligands and enzymes required for the post-translational processing of E-selectin, which are both associated with late recurring breast cancer. E-selectin inhibitor reduced the number of tumor cells homing to the niche. Whilst this suggests that the niches maintain dormancy, the functional role of E-selectin remains to be determined [218].

The endosteal niche is also important in dormancy. In a myeloma model, the use of labeling with the lipophilic tracer, DiD dye, to distinguish between proliferating and non-proliferating cells demonstrated that DiD dye-retaining DTCs persisted at the endosteal niche. The cells were also arrested in G0, did not express Ki67, displayed a quiescence-associated genetic profile, and were resistant to the chemotherapy, confirming a dormant phenotype. This effect is reversible as removal from the niche resulted in increased growth when grown in culture or re-injected to form tumors in naïve mice [219].

Direct interactions between the tumor cells and mesenchymal stromal cells at the niche resulted in maintained dormancy. Growth arrest specific 6 (Gas6) protein is expressed by both osteoblasts and fibroblasts [220] within the bone, and mediates interactions with tumor cells within the endosteal niche. Gas6 binds to receptor tyrosine kinases: Tyro3, MerTK, and Axl [221], of which the expression of the latter in prostate cancer cells is associated with increased tumor grade and bone metastasis but not lymph node metastasis [222]. Moreover, Axl is highly expressed by metastatic prostate cancer cells but not in non-metastatic cell lines [223,224]. Bone metastatic multiple myeloma cells also express Axl [219]. During colonization at the endosteal niche, the binding of annexin-II receptor on prostate cancer cells to annexin-II on osteoblasts induces the expression of Gas6 receptors. These bind to Gas6, which induces growth arrest and protection from apoptosis. Prostate cancer cells also exhibited decreased growth in a bone environment from Gas6^+/+^ animals in comparison to Gas6^−/−^ animals [225]. Together, this suggests that Gas6-Axl interactions at the endosteal niche can induce both quiescence and treatment resistance, and therefore dormancy, in prostate cancer [226]. A similar role of Gas6 at the endosteal niche is also demonstrated in leukemia, where Gas6 mediates migration and binding to MerTK, which protects the cancer cells from apoptosis, therefore inducing treatment resistance [103]. The equilibrium between the level of Axl and Tyro3 receptor expression mediates the proliferation status: high levels of Axl result in quiescence; high levels of tyro3 result in proliferation. Using a subcutaneous model of prostate cancer from which tumor cells disseminate to the bone, bone marrow DTCs exhibited reduced proliferation, which was associated with increased Axl and reduced tyro3 expression in comparison to the primary tumor. In contrast, the reduction of Axl expression resulted in the reactivation of proliferation and DTCs that developed into metastatic lesions exhibited decreased Axl, suggesting that Axl promotes dormancy [224]. Furthermore, hypoxia has been shown to induce dormancy [227], which may be due to the promotion of Gas6-Axl interactions. Through self-regulatory mechanisms, the binding of Gas6 to Axl results in the downregulation of Axl in prostate cancer cells in vitro. Hypoxic conditions, mimicked using CoCl2, prevented the Gas6-mediated downregulation of Axl and therefore can stabilize Gas6-Axl interactions [223]. The bone marrow, particularly the HSC niche, is a hypoxic microenvironment [42,43,44,45], with HIF1α playing a major regulatory role in HSC maintenance and quiescence [228]. The hypoxic bone marrow may therefore stabilize Gas6-Axl interactions between osteoblasts and prostate cancer cells, maintaining long-term adhesion to the niche and dormancy.

As discussed previously, DTCs colonized to the endosteal niche interact with OPN, which also maintains dormancy as quiescence is induced within these tumor cells [104], similar to the maintenance of quiescent HSCs [69,100]. Dormant cells also localize to OPN at the osteoblastic niche in a model of acute lymphoblastic leukemia (ALL). DiD dye-retaining ALL cells were identified at the OPN-rich trabecular bone surfaces in vivo and highly express several OPN receptors—including α4β1 and α5β1—that mediate binding. Antibody neutralization of OPN not only prevents this binding to the endosteal niche, but also reactivates the cell cycle in ALL and increases bone metastasis burden. The disruption of the OPN interactions also sensitizes ALL cells to treatment as the co-treatment of chemotherapy with OPN neutralization was more effective than chemotherapy alone, and may serve as a therapeutic potential [104]. OPN may also be important in the dormancy of DTCs from solid tumors. Whilst some studies have shown that OPN promotes tumor proliferation in solid tumors, this is often at the primary site [32]. Furthermore, ALL cells bind to thrombin-cleaved OPN and weakly to full-length OPN, which may explain the different effects on tumor growth observed between ALL and primary tumors. Thrombin-cleaved OPN has a specific epitope recognized by the α4β1 and α9β1 integrins, and therefore may be a specific mechanism in the bone microenvironment [104].

The CXCL12-CXCR4 interactions at the endosteal niche appear to be important in maintaining dormancy and treatment resistance by maintaining the cancer cells in the G0 phase of the cell cycle. CXCR4 inhibition using AMD3100 to disrupt the adhesion of multiple myeloma cells to the niche mobilized the tumor cells into the circulation, where they were sensitive to anti-tumor treatments including doxorubicin and bortezomib. This resulted in a significant reduction in bone metastatic burden in animals treated with a combination of AMD3100 and bortezomib, compared to bortezomib alone [105]. A similar mechanism was also shown to sensitize acute myeloid leukemic cells to chemotherapy [106], and may serve as a therapeutic potential. The expression of CXCR4 is also upregulated in patients with late-recurring breast cancers [218], further supporting its involvement in dormancy.

Dormancy within the bone marrow may also be regulated by transforming growth factor beta 2 (TGFβ2) signaling. In a model of HNSCC metastasis, DTCs present after resection of a primary subcutaneous tumor begun aggressive proliferation and developed metastasis in the lungs, where TGFβ2 is low. In contrast, DTCs remained dormant in the bone marrow due to increased TGFβ2 signaling. This could be prevented by TGFβ2 inhibition, and may therefore serve as a mechanism to reactivate tumor cells and prevent dormancy. However, whether this makes them more sensitive to therapeutic agents needs to be determined [107]. Whilst the source of TGFβ2 was not investigated, it is expressed by several stromal cells including MSCs, fibroblasts, and osteoblasts [115,139,229]. Stromal-derived TGFβR1 may also mediate dormancy in breast cancer. A co-culture of breast cancer cells with mesenchymal stromal cells including MSCs and osteoblasts in a 3D biomatrix resulted in cell cycle arrest and the decreased proliferation of the breast cancer cells. This was prevented by the inhibition of TGFβR1 as well as other cytokine receptors VEGFR and PDGFRβ [230]. BMP, which is a member of the TGFβ family, may play a role in maintaining dormancy by inhibiting the ability of the cancer cells to self-renew. Within a bone metastatic model of prostate cancer, systemic treatment of BMP7 prevented the outgrowth of tumor cells and maintained a dormant phenotype [231]. BMP7 is secreted from bone stromal cells and induces a quiescent phenotype in prostate cancer cells via the binding of BMPR2, of which the expression level is inversely correlated with bone metastasis recurrence. BMP7 treatment results in decreased tumor growth both in vitro and in vivo [231]. Similarly, within a model of breast cancer lung metastasis, stroma-derived BMP inhibited the outgrowth of disseminated tumor cells. The overexpression of Coco, an antagonist of BMP4, reactivates dormant breast cancer cells within lung metastasis by inducing proliferation, whereas control cells remained quiescent. Of note, Coco had no effect on primary tumor growth, suggesting that the effect is specific to the secondary site [108]. Whilst BMP treatment may maintain dormancy, inhibition may reactivate the cells or prevent dormancy.

Another cytokine involved in maintaining survival at the HSC niche is bone marrow stromal cell-derived fibroblast growth factor (FGF). Basic FGF is secreted by both fibroblasts and endothelial cells and inhibits the proliferation of MCF-7 breast cancer cells by inducing the balance of mitogenic and inhibitory signals towards a net effect where the cell cycle accumulates in G0 [232]. This induces the expression of α5β1 integrin in breast cancer cells to mediate binding to fibronectin in the ECM and leads to survival signaling through the phosphoinositide 3-kinase (PI3K)/Akt pathway [233].

Gap junction intercellular communication between cancer cells and bone marrow stroma also mediates dormancy by facilitating the transport of molecules that induce quiescence. A co-culture of breast cancer cells with bone marrow cells from patients resulted in decreased tumor cell growth correlated with the formation of gap junctions composed of connexion-43 [234]. The formation of gap junctions between osteoblasts and melanoma cells has also been observed [166]. Gap junctions serve as a transport system for microRNAs (miRNAs) from stromal cells to breast cancer cells, which can induce quiescence and cell cycle arrest in cancer cells. Many of the miRNAs, such as 127, 197, 222, and 223, are CXCL12-dependent, and therefore may be induced during the homing mechanism [109,110]. Dormancy-inducing miRNAs from mesenchymal stromal cells can also be transported to tumor cells via the formation of exosomes. During a co-culture of bone marrow MSCs with a bone metastatic breast cancer cell line, MSC-derived exosomes induced a dormant phenotype characterized by cell cycle arrest at G0 and resistance to chemotherapy. This was due to the transport of miR23b in the exosome that targets and decreases myristolyated alanine-rich C-kinase substrate (MARCKS), which is involved in the promotion of cell cycling. Notably, cancer cells isolated from bone marrow of patients exhibit increased miR23b and decreased MARCKS expression in comparison to cancer cells isolated from the primary breast tumor, suggestive a mechanism specific to the bone metastatic phenotype [111]. MSC-derived miRNA-222/223 transported by exosomes also induces dormancy in breast cancer. Interestingly, exosomes from breast cancer-primed MSCs contained more miRNA-222/223 and were more effective in inducing dormancy compared to naïve MSCs, suggesting that breast cancer must stimulate the MSCs. Furthermore, the effects were not observed using a low metastatic line. Of most significance, targeting these miRNAs reversed the dormant phenotype and enhanced their sensitivity to chemotherapy, highlighting a therapeutic potential that may be more specific to the tumor cells and therefore less toxic [235]. There is increasing evidence for a role of microRNA (miRNA) throughout the establishment of bone metastasis [236,237], including the induction of dormancy; therefore, this may also be an effective therapeutic target.

### 4.2. Reactivation from Dormancy

The mechanisms that govern the activation of dormant cells remain to be fully elucidated. As dormant cells do not proliferate, reactivation is less likely to be a result of genetic adaptations, possibly due to non-genetic mechanisms such as molecular interactions with the bone microenvironment [238].

The reactivation of dormant cells may occur due to the disruption of the molecular interactions involved in the maintenance and induction of dormancy discussed above. For example, dormancy can be targeted by disrupting the interactions at the niche or using CXCL12 inhibitors and G-CSF, which cause the mobilization of DTCs from the niche into the circulation and re-sensitize them to chemotherapy [104,239]. Whilst many studies describe how DTCs escape dormancy after these treatments, the endogenous mechanisms involved in spontaneous reactivation from dormancy have not been addressed. Although the perivascular niche is thought to maintain dormancy, newly formed vasculature, which is rich in TGFβ and periostin, induces outgrowth of the breast cancer. This may serve as the point at which the dormant DTCs begin to proliferate [212]. Components of the ECM, which are secreted from stromal cells, may play a role in reactivation. Fibronectin is associated with reactivation, and has been shown to be regulated through urokinase plasminogen activator receptor (UPAR), α5β1, ERK, and p38 [240]. In a model of breast cancer lung metastasis, dormant tumor cells injected into the circulation interacted with collagen I in the lung via binding to the integrin β1, which activated Src and focal adhesion kinase (FAK) and subsequently induced the proliferation and growth of the metastatic lesion. This effect was reversed by targeting components of this pathway. TGFβ1 treatment also promoted growth and metastasis by increasing collagen I deposition [241]. Collagen [219] or TGFβ1 [115,139,229] secreted from mesenchymal stromal cells may have similar effects in bone metastasis. Osteoclast-derived collagen I also induces reactivation in multiple myeloma by remodeling the endosteal niche [219].

Remodeling of the bone by osteoclasts may also reactivate dormant cells in breast cancer [219]. The expression of VCAM1 is associated with early recurrence and was essential for escape from dormancy and the acquisition of osteolytic bone metastatic ability. This was due to the VCAM1-induced activation and recruitment of osteoclasts via binding to the osteoclast-expressed integrin α4β1. Dormant DTCs acquire VCAM1 expression whilst in the bone marrow, partially dependent on nuclear factor kB (NF-kB) signaling. The activation of the osteoclasts also initiated the osteolytic cycle in bone metastasis, therefore promoting tumor growth, as discussed later. Both the inhibition of VCAM1 and integrin α4 attenuated bone metastasis progression [242]. The role of osteoclasts in reactivation was further supported by Lawson et al. [219], who demonstrated in in vitro cultures that macrophages used to mimic osteoclasts promoted proliferation, and RANKL-induced increase in osteoclasts resulted in decreased dormant cells within the bone marrow [219].

## 5. Mesenchymal Stromal Cells in Bone Metastatic Growth

**Figure 3:**
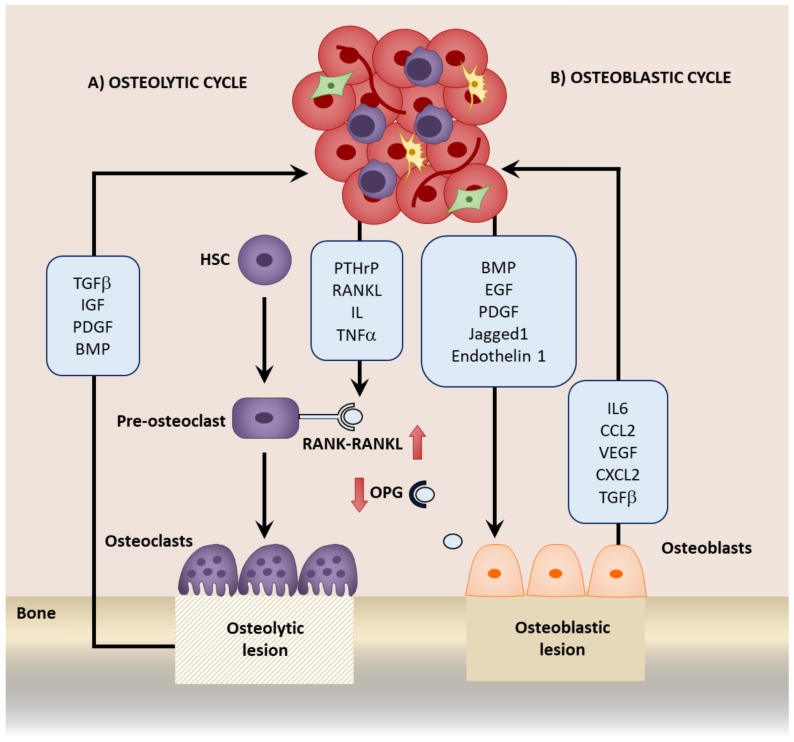
Disruption of bone turnover in an (**A**) osteolytic and (**B**) osteoblastic bone metastasis, and growth factors involved [5,27,92,112,114,115,116,117,118,243,244,245].

Once reactivated from dormancy, tumor cells proliferate, developing into a macrometastasis. As the metastasis grows, the bone remodeling process is disrupted. Bone turnover is regulated by the balance between osteoclast-mediated osteolysis, or bone resorption, and osteoblast-mediated osteogenesis, or bone production. The presence of bone metastasis disturbs this balance, resulting in a bone metastatic lesion. These lesions can be either osteolytic, which is often observed in breast cancer and manifests as a loss of bone, or osteoblastic, which is common in prostate cancer and causes sclerosis of the bone (Figure 3) [5].

In the presence of an osteolytic bone metastasis, the tumor cells secrete osteoclast-activating factors including PTHrP, RANKL, interleukins, and TNFα, and reduce the activity of OPG which further activate the RANK-RANKL pathway. This enhances osteolysis and consequently growth factors, such as TGFβ, insulin-like growth factor (IGF), platelet-derived growth factor (PDGF), and BMP, are released from the bone matrix, further promoting tumor growth and inducing a “vicious” cycle as the metastasis grows [5,114]. One major predominant loop in this cycle is between TGFβ and PTHrP. TGFβ released from the bone further induces PTHrP from the tumor cells, which in turn induces RANKL and therefore promotes osteoclastogenesis [112,243]. The inhibition of TGFβ1 results in a reduction of the incidence of bone metastasis, and may serve as a potential therapy [243,244,245]. Interactions between stromal cells and tumor cells can also promote osteoclast activity, including VCAM1-α4β1 interactions in myeloma [184] and breast cancer [242]. Inhibiting these interactions also prevents bone metastasis recurrence [27,242].

In osteoblastic bone metastasis, this cycle is accompanied by the release of factors from tumor cells, such as BMP, EGF, and PDGF, that stimulate osteoblasts. In turn, growth promoting factors including IL6, CCL2, VEGF, and CXCL2 are released from the osteoblasts, which promote tumor cell growth, again initiating an additional cycle [27,114]. PTH released during this misbalance of bone turnover has been shown to enhance tumor growth by increasing the osteoblasts in a mouse model of breast cancer [27] and myeloma-bearing rats [246]. Prostate cancer bone metastatic cells also express increased PTH receptor, to which the binding of PTH promotes bone metastatic growth [247]. Moreover, within a model of bone metastasis established by intratibia injection of prostate cancer cells, a paracrine interaction between the cancer cells and osteoblasts exists where prostate cancer-derived PTHrP induces CCL2 secretion in the osteoblasts. This subsequently induces the growth of tumor cells. CCL2 inhibition attenuated growth in the bone, and was associated with decreased bone resorption [92]. In breast cancer bone metastasis, tumor-derived Jagged1 induces IL6 and TGFβ secretion from osteoblasts, which not only promotes tumor cell proliferation directly, but also induces osteoclasogenesis and therefore osteolysis, resulting in a further release of TGFβ. Furthermore, TGFβ induces Jagged1, therefore establishing a positive feedback to maintain growth [115]. Endothelin-1 is also secreted from tumor cells and activates osteoblasts by downregulating Wnt inhibitor Dickkoft-1 (Dkk1). Endothelin-1 inhibitors provide a therapy to target bone metastasis growth [116,117,118]. In the bone metastasis of multiple myeloma, MSC-derived factors induced the secretion of Dkk1 from myeloma cells that prevented MSC differentiation to osteoblasts. The undifferentiated MSCs expressed IL6 that increased the growth of the myeloma cells, which subsequently produced more Dkk1, establishing a positive feedback loop. This could be disrupted by using either an IL6 inhibiting antibody or a Wnt activator, highlighting potential therapeutic mechanisms to prevent bone metastatic lesion growth [119]. Interactions with the niche may also mediate growth. For example, OPN mediates the in vitro growth of prostate cancer cells [196] and binds to β3 integrins to enhance the in vivo growth of primary breast cancer xenografts [189]. Annexin-II may also promote growth via the mitogen-activated protein kinase (MAPK) pathway [155].

Other MSC- and CAF-derived growth factors are also involved in the growth of tumor cells in many different primary cancers (Table 2), including breast [248,249], lung [250], prostate [33,229], and melanoma [250,251]. Similar growth factors may be involved in bone metastasis. Although stromal-derived CXCL12 is implicated in the growth of several cancers, it may also be involved in dormancy as discussed previously [105]. These functional differences may be due to the Src-status of the tumor cells, which mediates responses to CXCL12 produced by the bone microenvironment. CXCL12-CXCR4 interactions activate Akt signaling in the cancer cells to promote survival, which is potentiated by Src. Src-depleted breast cancer cells exhibited attenuated growth within the bone, which was restored by Src expression. Furthermore, Src is associated with relapse in the bone, but not other organs, suggesting that it is necessary for the survival and outgrowth of latent bone metastasis [252]. Cells expressing high Src may be selected for in the primary tumor. CAFs secrete CXCL12 and IGF1 to select for hyperactive Src clones, which are highly metastatic to the bone. This means that once these cells reach the bone marrow they are selected to survive in the CXCL12 environment [40].

Angiogenesis is also important for metastatic growth to ensure the delivery of nutrients and oxygen, particularly as the tumor size increases [269], and is promoted by paracrine interactions between mesenchymal stromal cells and tumor cells. In the bone metastasis of prostate cancer, tumor-derived PTHrP induces CCL2 from osteoblasts, which acts on the tumor cells to induce VEGF secretion and therefore promotes angiogenesis [92]. Although further research into the effect of mesenchymal stromal cells on angiogenesis in bone metastasis is required, other MSC- and CAF-derived factors enhance angiogenesis in primary tumors, including VEGF, TGFβ, CXCL12, IL6, IL8, and HGF [270,271,272,273,274]. Many of these factors, for example CXCL12, TGFβ1, and IL6, are upregulated in bone metastasis [275], and therefore may play a similar role in promoting angiogenesis and the growth of bone metastasis. As well as having direct effects on endothelial cells, including migration and tube formation, MSCs may also promote angiogenesis by recruiting M2 macrophages, which secrete angiogenic cytokines such as IL6, IL8, and VEGF [276]. Crosstalk between mesenchymal stromal cells and the immune cells within the TME is also important for creating an immunosuppressive environment that permits tumor growth. To achieve the immunosuppression, mesenchymal stromal cells can express factors that manipulate different aspects of the immune system including: a decrease in CD8^+^ T cells and NK cells; an increase in immunoregulatory CD4^+^ T cells accompanied by increased Th2 and decreased Th1 cytokines [251]; CCL2-CCR2, IL6, and IL10-mediated infiltration and polarization of macrophages into a alternatively activated, tumor-promoting phenotype [57,195]; and the CXCL1/CXCL2/CXCL5-CXCR2-dependent recruitment of tumor-suppressive neutrophils [58]. The interactions between mesenchymal stromal cells and the immune system are complex and more information is reviewed in References [277,278]. Moreover, most studies are on primary tumors, and thus research on the immune-modulatory effects of mesenchymal stromal cells in the context of bone metastasis is required.

## 6. Mesenchymal Stromal Cells in Treatment Resistance

Resistance to anti-tumor treatments is a major challenge for patients with bone metastasis, and current treatment only involves palliative therapy to prevent further bone loss and treat associated symptoms. As discussed, treatment resistance may be induced due to the dormant, non-proliferating phenotype maintained by the mesenchymal stromal cells at the niche. Targeting the pathways discussed may provide an adjuvant therapy to treat bone metastasis.

Chemotherapy treatment can also promote resistance by stimulating survival factors from stromal cells. For example, the treatment of bone lesions with chemotherapy induced Jagged1 expression in osteoblasts, which promote the survival and resistance of the breast cancer cells. In addition, osteoblast-derived Jagged1 enhanced tumor seeding, suggesting that chemotherapy may enhance bone metastasis by enhancing colonization. The inhibition of Jagged1 sensitized bone metastasis to chemotherapy and prevented relapse, thus co-treatment of chemotherapy with Jagged1 inhibitors may improve clinical outcome. Whilst there are very few studies investigating the role of mesenchymal stromal cells in the treatment resistance of bone metastasis, the molecular mechanisms involved in the stromal-induced resistance of primary tumors may offer insight. Stromal secreted factors that promote resistance include platinum-induced fatty acids in ovarian cancer [279], IL8 in doxorubicin-treated breast cancer [280], WNT16B in prostate cancer treated with chemotherapy [281], and fibronectin-α5β1 integrin interactions in doxorubicin-treated multiple myeloma [282]. Further information can be reviewed in Reference [279].

## 7. Conclusions

Although the role of mesenchymal stromal cells in the development of primary tumors has been extensively researched, their contribution to bone metastasis remains to be fully elucidated. It is clear that the TME in bone metastasis is different from its primary tumor counterpart. Studies demonstrate that MSCs, fibroblasts, and osteoblasts play a role in tumor cell homing and colonization to the bone. The DTCs can become dormant, but can also be reactivated and begin to grow, eventually establishing as a bone metastatic lesion. Further understanding of the role of mesenchymal stromal cells in bone metastasis is required; however, there are several obstacles that need to be overcome. Firstly, the true identification of MSCs, pericytes, and fibroblasts, including the similarities between these cells, needs to be further clarified. Furthermore, the use of MSCs in vitro requires more consistency between studies, and standardized isolation and culturing techniques should be established. In vivo models will be key in further investigating the role of stromal cells in bone metastasis, and preclinical models to mimic clinical bone metastasis more accurately must be developed. This includes spontaneous metastasis from primary tumors to the bone with a latent period between resection or treatment of the primary tumor and recurrence. Current experimental models are often aggressive and do not allow the metastatic niche during the latent or dormant period to be studied. Moreover, the processes such as homing and colonization may be transient and rare events. Live imaging techniques will therefore be essential in capturing these processes. Identifying the responsible mechanism is further complicated by the complexity of the TME with many types of stromal cells contributing to each stage, and must be considered together when researching their role in bone metastasis.

Nevertheless, the molecular interactions between mesenchymal stromal cells and tumor cells may serve as potential therapeutic targets. Treatments targeting the stromal cells within tumors may be less likely to exhibit resistance as the stromal cells are genetically stable in comparison to tumors cells in which genetic mutations can induce drug resistance. However, the overlap between the mechanisms involved in physiological processes such as HSC maintenance and those involved in bone metastasis may cause adverse side effects if therapeutically targeted. Elucidating the molecular interactions between the tumor and stromal cells unique to bone metastasis may allow the development of more specific drugs to treat bone metastasis. Theoretically, bone metastasis may be treated as a chronic disease if the dormant phenotype of DTCs can be maintained. Alternatively, adjuvant therapies at the time of primary tumor treatment may reactivate the DTCs, causing their mobilization and sensitization to treatment. Further research into the molecular mechanisms specific to bone metastasis is essential to identify the most appropriate pathway and process to target in order elucidate new therapies to overcome this disease.

## Figures and Tables

**Table 1:** Stromal-derived chemoattractants mediating tumor cell migration.

Chemotactic Molecule	Cancer Type
CXCL12-CXCR4	Melanoma [82] and breast cancer [122] bone metastasis
ILβ1 and leptin	Breast cancer bone metastasis [137]
CXCL12-CXCR4/CXCR7	Osteosarcoma [138]
CXCL1- and CXCL5-CXCR4	Breast cancer [139]
TGFβ	Breast [139] and hepatocellular cancer [140]
CCL5- and CCL9-induced MMP	Breast cancer [141]
CCL5-induced collagen	Tongue cancer [142]
CCL5	Osteosarcoma cells [143]
VEGF-CXCR4	Osteosarcoma [144]
IL8	Gastric [145]
IL6	Ovarian [146]
CXCL12	Melanoma [147]
CCL5 mediated by tumor-derived OPN	Breast cancer [32]

**Table 2:** Mesenchymal stromal cell-derived factors involved in tumor growth.

Mesenchymal Stromal Cell Source	Cancer Type	Growth Factor
**MSCs**	Breast	CCL5 [32]
	Breast	IL6 [253]
	Breast	CXCL7 [249]
	Breast	CXCL12–CXCR4 [254,255]
	Breast	Collagen-DDR2 [256]
	Prostate	TGFβ [229]
	Colorectal	Galectin 3 [257]
	Neuroblastoma, osteosarcoma, colorectal	IL6 [258,259,260,261]
**MSC-derived CAFs**	Ovarian	IL6, HGF, EGF [262]
	Gastric	IL6, Wnt, BMP [263]
	Liver	miR155 [264]
	Breast	CXCL12 [60]
**CAFs**	Breast	CXCL12-CXCR4 [265,266]
	Breast	VEGF [267]
	Breast	HGF [268]
**Osteoblast**	Prostate bone metastasis	CCL2 [92]

## Abbreviations

ALL	acute lymphoblastic leukemia
αSMA	alpha-smooth muscle actin
BDNF	brain-derived neurotrophic factor
BMP	bone morphogenetic protein
BSP	bone sialoprotein
CAF	cancer-associated fibroblasts
CCL	C-C motif chemokine
CTC	circulating tumor cell
CXCL	C-X-C motif chemokine 12
CXCR	C-X-C chemokine receptor
DDR	discoidin domain receptor family member 1
DKK1	dickkopf-related protein 1
DTC	disseminated tumor cell
ECM	extracellular matrix
EGF	epidermal growth factor
EPO	erythropoietin
ERK	extracellular receptor kinase
FAK	focal adhesion kinase
FAP	fibroblast-activated protein
FGF	fibroblast growth factor
FSP	fibroblast specific protein (S100A4)
Gas6	growth arrest specific 6
HBGF	heparin-binding growth factor
HGF	hepatocyte growth factor
HIFα	hypoxia-inducible factor
HPC	hematopoietic progenitor cell
HSC	hematopoietic stem cell
ICAM1	intercellular adhesion molecule 1
IGF	insulin-like growth factor
IL	interleukin
MAPK	mitogen-activated protein kinase
MARCKS	myristolyated alanine-rich C-kinase substrate
miRNA	microRNA
MMP	matrix metalloproteinase
MSC	mesenchymal stem cell
NF-kB	nuclear factor kB
NG2	neuron-glial antigen 2 (CSPG4)
OPG	osteoprotegerin
OPN	osteopontin
PDGF	platelet-derived growth factor
PDGFRβ	platelet-derived growth factor receptor beta
PI3K	phosphoinositide 3-kinase
PTH	parathyroid hormone
PTHrP	parathyroid hormone-related protein
RANK/RANKL	receptor activator of nuclear factor kappa-B/ligand
Runx2	runt-related transcription factor 2
sRAGE	soluble receptor for advanced glycation end products
SOD2	superoxide dismutase 2
TGFβ	transforming growth factor beta
TME	tumor microenvironment
TNFα	tumor necrosis factor alpha
VCAM1	vascular cell adhesion protein 1
VEGF	vascular endothelial growth factor
